# Exposure to Traffic Pollution and Increased Risk of Rheumatoid Arthritis

**DOI:** 10.1289/ehp.0800503

**Published:** 2009-03-04

**Authors:** Jaime E. Hart, Francine Laden, Robin C. Puett, Karen H. Costenbader, Elizabeth W. Karlson

**Affiliations:** 1 Channing Laboratory, Department of Medicine, Brigham and Women’s Hospital and Harvard Medical School, Boston, Massachusetts, USA; 2 Department of Epidemiology, Harvard School of Public Health, Boston, Massachusetts, USA; 3 Exposure, Epidemiology, and Risk Program, Department of Environmental Health, Harvard School of Public Health, Boston, Massachusetts, USA; 4 South Carolina Cancer Prevention and Control Program, University of South Carolina, Columbia, South Carolina, USA; 5 Departments of Environmental Health Sciences and Epidemiology and Biostatistics, Arnold School of Public Health, University of South Carolina, Columbia, South Carolina, USA; 6 Division of Rheumatology, Immunology, and Allergy, Brigham and Women’s Hospital and Harvard Medical School, Boston, Massachusetts, USA

**Keywords:** air pollution, prospective study, rheumatoid arthritis, roadway proximity, traffic

## Abstract

**Background:**

Rheumatoid arthritis (RA) is a chronic systemic inflammatory disease that affects approximately 1% of the adult population, and to date, genetic factors explain < 50% of the risk. Particulate air pollution, especially of traffic origin, has been linked to systemic inflammation in many studies.

**Objectives:**

We examined the association of distance to road, a marker of traffic pollution exposure, and incidence of RA in a prospective cohort study.

**Methods:**

We studied 90,297 U.S. women in the Nurses’ Health Study. We used a geographic information system to determine distance to road at the residence in 2000 as a measure of traffic exposure. Using Cox proportional hazard models, we examined the association of distance to road and incident RA (1976–2004) with adjustment for a large number of potential confounders.

**Results:**

In models adjusted for age, calendar year, race, cigarette smoking, parity, lactation, menopausal status and hormone use, oral contraceptive use, body mass index, physical activity, and census-tract-level median income and house value, we observed an elevated risk of RA [hazard ratio (HR) = 1.31; 95% confidence interval (CI), 0.98–1.74] in women living within 50 m of a road, compared with those women living 200 m or farther away. We also observed this association in analyses among nonsmokers (HR = 1.62; 95% CI, 1.04–2.52), nonsmokers with rheumatoid factor (RF)-negative RA (HR = 1.77; 95% CI, 0.93–3.38), and nonsmokers with RF-positive RA (HR = 1.51; 95% CI, 0.82–2.77). We saw no elevations in risk in women living 50–200 m from the road.

**Conclusions:**

The observed association between exposure to traffic pollution and RA suggests that pollution from traffic in adulthood may be a newly identified environmental risk factor for RA.

Rheumatoid arthritis (RA) is a chronic systemic inflammatory disease affecting approximately 1% of the adult population (Doran et al. 2002; Drosos et al. 1997; Gabriel et al. 1999). Genetic factors are thought to be responsible for < 50% of RA risk, suggesting that environmental factors could contribute to the development of RA in the genetically predisposed (Begovich et al. 2004; Gregersen et al. 1987; Kobayashi et al. 2005). Cigarette smoking is a strong environmental risk factor for the development of RA, with a clear dose–response relationship. Risk of RA rises after 10 pack-years of smoking and remains elevated up to 20 years after smoking discontinuation (Costenbader et al. 2006). Epidemiologic evidence has also suggested associations of RA with occupational exposures to silica and mineral oil and with environmental exposures such as cigarette smoke (Criswell et al. 2002; Hazes et al. 1990; Heliovaara et al. 1993; Hernandez Avila et al. 1990; Karlson et al. 1999; Krishnan et al. 2003; Padyukov et al. 2004; Raychaudhuri et al. 2008; Stolt et al. 2003, 2005; Sverdrup et al. 2005; Symmons et al. 1997; Uhlig et al. 1999; Vessey et al. 1987; Voigt et al. 1994), suggesting that respiratory exposures activating the immune system may lead to RA. We have recently examined the geographic variation of RA in a prospective cohort of U.S. women (Costenbader et al. 2008). We observed significantly higher RA risk in the Midwest and Northeast regions of the United States (compared with the West) in women who had lived in one region throughout most of their lives. These regions have historically had higher levels of air pollution, and our evolving understanding of RA pathogenesis suggests that inhaled particulate matter, similar to cigarette smoke, may induce local lung inflammation as well as systemic inflammation. Indirect support of this hypothesis comes from the observation that air pollution has been clearly linked with other diseases of local lung and systemic inflammation, including asthma and chronic bronchitis, cardiovascular disease, and lung and laryngeal cancers (Dockery et al. 1993; Karakatsani et al. 2003; Künzli et al. 2005; Laden et al. 2006; Nafstad et al. 2003; Penard-Morand et al. 2005; Pereira et al. 2005; Pope et al. 2002; Pope and Dockery 2006; Sunyer 2001; van Eeden et al. 2005). Associations with overall mortality have been shown most strongly with particles from traffic, coal, and residual oil combustion (Laden et al. 2000; Schwartz et al. 2002). Therefore, in this analysis we examine the association of incidence of RA and distance to the nearest road as a marker of traffic pollution in a cohort of U.S. women.

## Materials and Methods

### Study population

The Nurses’ Health Study (NHS) is a long-term prospective cohort study of U.S. female nurses. The NHS was initiated in 1976 when 121,700 U.S. registered female nurses, 30–55 years of age, completed a mailed questionnaire and provided informed consent. At the study inception they resided in 11 states throughout the United States (California, Connecticut, Florida, Massachusetts, Maryland, Michigan, New Jersey, New York, Ohio, Pennsylvania, and Texas). Although most of the nurses have not moved, they now reside in all 50 states. Follow-up questionnaires, with response rates > 90%, are mailed every 2 years to update information on risk factors and the occurrence of major illnesses. We included women in the present study if their 2000 residential address was successfully geocoded to the street segment level in the continental United States and they had no history of RA or cancer (other than nonmelanoma skin cancer) at baseline in 1976. A total of 90,297 members were available for analysis.

### Assessment of outcome

We used a two-stage procedure to identify nurses with RA. All nurses reporting RA or other connective tissue disease on a biennial questionnaire (*n* = 11,674) received a follow-up screening questionnaire for connective tissue symptoms (Karlson et al. 1995). If the screening questionnaire was positive, we requested medical records, which underwent detailed examination for American College of Rheumatology diagnostic criteria for RA. Subjects who self-reported but later denied RA diagnosis, denied permission to obtain medical records, or who had a negative screening questionnaire were excluded (*n* = 8,877) (Karlson et al. 2004). In this analysis, we included a total of 687 incident cases (1976–2004), 390 (57%) of whom had rheumatoid factor (RF)-positive RA as determined by medical record review.

### Exposure assessment

We studied distance to road in the year 2000 as a proxy for traffic pollution exposure. Distance to road (in meters) for all available nurses’ addresses was determined using geographic information system software (ArcGIS, version 9.2; ESRI, Redlands, CA). Road segments in the 2000 U.S. Census Topologically Integrated Geographic Encoding and Referencing system (TIGER) files (http://www.census.gov/geo/www/tiger/) were selected by U.S. Census feature class code to include A1 (primary roads, typically interstates, with limited access), A2 (primary major, noninterstate roads), or A3 (smaller, secondary roads, usually with more than two lanes) road segments. We calculated the shortest distance between each address and the closest road segment. We conducted analyses using the distance to the closest of all three road types, and the distance to the nearest primary road (A1, A2 only). For the main analyses, we used the distance to A1–A3 roads at the 2000 mailing address. In sensitivity analyses, we also assessed the associations with distance to road for all other available address years and to primary roads only. Based on the distribution of distance to road in this cohort and previous exposure studies showing exponential decay in exposures with increasing distance to road, we divided distance to road into the following categories (0–50, 50–200, ≥ 200 m) (Adar and Kaufman 2007; Lipfert et al. 2006; Lipfert and Wyzga 2008; Sahlodin et al. 2007; Zhu et al. 2002).

### Additional covariates

Information on potential confounders and effect modifiers is updated every 2 years in the NHS, so we assigned each woman updated covariate values for each individual questionnaire cycle where appropriate. We examined possible confounding by age (in months), non-Caucasian race, age at menarche, parity, total months of lactation, current menopausal status, menopausal hormone use, oral contraceptive use, physical activity, and body mass index (BMI), many of which have been shown to be risk factors for RA (Costenbader et al. 2008; Karlson et al. 2004). To control for smoking, we used data from lifetime smoking history to calculate pack-years (number of packs/day multiplied by number of years of cigarette smoking) and current smoking status (current/former/never). Because smoking is a major risk factor for RA and residual confounding by smoking may obscure any traffic pollution effect, we also restricted models to never-smokers. To control for individual level socioeconomic status, we included several variables, including nurses’ educational level, occupation of both parents, marital status, and, if applicable, husband’s education. To control for area-level socioeconomic status, we included area-level information from the 2000 Census on tract level median income and house value.

### Statistical analysis

Time-varying Cox proportional hazards models were used to assess the relationship of incident RA (1976–2004) with distance to road. These models were based on a biennial time scale and were used to estimate hazard ratios (HRs) and 95% confidence intervals (CIs). Person-time accrued from 1 January 1976 until diagnosis of RA, date of death, or the end of follow-up (31 May 2004), whichever was first. All Cox models were stratified by age in months and calendar year. We also ran sensitivity analyses restricted to women who were nonsmokers at baseline and by RF status (positive/negative). All statistical analyses used SAS (version 9.1; SAS Institute Inc. 2006).

## Results

Table 1 presents the characteristics of the cohort at baseline (1976) by distance to road category. The average (± SD) age at baseline of women in NHS was 42.4 ± 7.1, and the average BMI was 23.7 ± 4.0. Only 31.4% of the women were current smokers, and 44.4% had never smoked cigarettes. Most of the cohort was premenopausal, and more than half of the postmenopausal women had never used postmenopausal hormones. Twenty percent of the women had < 3 metabolic equivalent hours per week of physical activity, and only 11.2% had more than 27. Most of the women had Registered Nurse degrees, were married, and had husbands with a high school education or greater. There was little difference in the distribution of RA risk factors by exposure level. As expected, members of the cohort living closer to roads did live in census tracts with lower median family income and home values than did those living farther away from roads.

In models adjusted for age and calendar year, individuals living within 50 m of an A1–A3 road had a 33% (95% CI, 0–77%) higher risk of incident RA compared with those living 200 m or farther away (Table 2). Women living 50–200 m from these roads had a non-statistically significant decreased risk (HR = 0.85; 95% CI, 0.66–1.09). Additional adjustment for race, cigarette smoking, parity/lactation, menopausal status and hormone use, oral contraceptive use, BMI, physical activity, and census-tract-level median income and house value did not change the results (0–50 m: HR = 1.31; 95% CI, 0.98–1.74; 50–200 m: HR = 0.84; 95% CI, 0.65–1.08). Among never-smokers, the association of living within 50 m and incident RA was slightly elevated compared with that in the full cohort, with an HR = 1.62 (95% CI, 1.04–2.52) in fully adjusted models. The results were similar in sensitivity analyses using a subsample of women who did not move between 1988–2004 [Supplemental Material, Table 1 (available online at http://www.ehpon-line.org/members/2009/0800503/suppl.pdf)], as well as analyses using distance to road in all other available address years (data not shown). This is reasonable given the residential stability in this cohort (58% of the cohort had only one address between 1988–2004, and among the participants who did move, the median number of moves was 1).

We also observed associations with distance to road with RF-positive RA (Table 3). In fully adjusted models, women living within 50 m of a road had a 44% higher risk (95% CI, 0–107%) of RF-positive RA than did those living 200 m or farther away. The association among never-smokers was similar but did not achieve statistical significance. Among RF-negative RA cases, women living within 50 m of a road had a non-statistically significant 15% increased risk (95% CI, –17% to 83%) in fully adjusted models. The association between distance to road and RF-negative RA among never-smokers was also elevated (HR = 1.77; 95% CI, 0.93–3.38).

In multivariable analyses restricted to primary (A1 and A2) roads (Table 4), women living within 50 m of a primary road had an HR of 1.63 (95% CI, 1.06–2.51), whereas those living 50–200 m away had an HR of 0.86 (95% CI, 0.60–1.23). Unlike models including A1–A3 roads, the HRs were not higher when the cohort was limited to nonsmokers. There were too few cases living within 50 m of a primary road to conduct analyses stratified by RF status.

## Discussion

In a prospective assessment of incident RA and distance to road, we found that women living within 50 m of an A1–A3 road are at a 31% increased risk of RA compared with women living more than 200 m away. This association was also observed in analyses among nonsmokers (especially among RF-negative cases) and in women with RF-positive RA. We found higher risks for those women living closer to primary roads (A1, A2). These results suggest that higher exposure to traffic pollution may be associated with RA risk. To the best of our knowledge, this is the first time an association had been demonstrated between RA incidence and residential distance to the closest road.

A large body of health effects literature has developed using distance to road as an indicator of traffic exposures (Adar and Kaufman 2007; Beelen et al. 2008; Brunekreef et al. 1997; Ciccone et al. 1998; Duhme et al. 1996; English et al. 1999; Finkelstein et al. 2004; Garshick et al. 2003; Guo et al. 1999; Hoek et al. 2002; Jerrett et al. 2005a, 2005b; Lipfert et al. 2006; Maheswaran and Elliott 2003; Nitta et al. 1993; Oosterlee et al. 1996; Tonne et al. 2007; Venn et al. 2000, 2001, 2005; Weiland et al. 1994; Wjst et al. 1993). Adverse health effects have been demonstrated with respiratory symptoms, asthma, lung function, and all cause and cardiopulmonary mortality in populations living near roads around the world. Because traffic is a major source of air pollution, these results are also consistent with the general air pollution health effect literature. Many studies have demonstrated the adverse effects of air pollution on mortality and morbidity (Downs et al. 2007; Pope and Dockery 2006; Schikowski et al. 2005; Sunyer et al. 2006), and specifically air pollution from traffic sources (Beelen et al. 2008; Laden et al. 2000; Schwartz et al. 2005). Our observed higher risks in nonsmokers are also consistent with results from the general air pollution literature. In an analysis of particulate matter exposures with all-cause mortality and coronary heart disease events in the NHS cohort, we found higher risks among women who were never-smokers, with no associations seen in current smokers (Puett et al. 2008). This suggests that the overall detrimental effects of smoking may mask the effects of traffic or air pollution. In these multivariable models, compared with never-smokers, the HR for being a current smoker was 2.12 (95% CI, 1.29–3.48) and for former smokers was 2.57 (95% CI, 1.62–4.09). Therefore, the 31% higher risk we observed for living within 50 m of an A1–A3 road, and the 63% higher risk for those near A1–A2 roads are much smaller than the risks from former/current smoking in this cohort.

Epidemiologic evidence has provided strong evidence that cigarette smoke is a risk factor for RA (Costenbader et al. 2006; Criswell et al. 2002; Hazes et al. 1990; Heliovaara et al. 1993; Hernandez Avila et al. 1990; Karlson et al. 1999; Krishnan et al. 2003; Padyukov et al. 2004; Stolt et al. 2003, 2005; Sverdrup et al. 2005; Symmons et al. 1997; Uhlig et al. 1999; Vessey et al. 1987; Voigt et al. 1994). Stronger effects have been seen in individuals with seropositive RA, similar to our findings with distance to road. Cigarette smoke contains hundreds of compounds, many of which have been shown to lead to elevated levels of inflammation (Bermudez and Ridker 2002; Tracy et al. 1997). Traffic exposures and cigarette smoking may act to increase risk of RA through similar mechanisms. Respiratory exposure to particulate matter (of which traffic is a major source) has similarly been associated with increased systemic inflammation (Jimenez et al. 2002; Peters et al. 1997; Seaton et al. 1995; van Eeden et al. 2005). Human exposure to high levels of ambient particles stimulates the production of inflammatory cytokines in the lung, which may stimulate the bone marrow to release neutrophils and monocytes into the circulation (Tan et al. 2000). In animals, respiratory exposure to particulate matter accelerates transit of neutrophils and monocytes in bone marrow and expands the leukocyte pool size (Mukae et al. 2001; Terashima et al. 1997). Studies in cellular and animal models suggest that reactive oxygen species are generated by respiratory exposure to particulate matter (Gonzalez-Flecha 2004). Reactive oxygen species and oxidative stress are generated indirectly by proinflammatory mediators released from stimulated macrophages, but there is also evidence of direct effects of particles on intracellular sources of reactive oxygen species. Many proinflammatory genes induced upon exposure to particulate matter are regulated by redox-sensitive transcription factors, such as nuclear factor κ B (NFκB) (Shukla et al. 2000). *In vivo* models of inhalation exposure to particulate matter demonstrate that NFκB- regulated genes, including interleukin-6, tumor necrosis factor-α, and γ-interferon, are up-regulated by exposure to particulate matter (Jimenez et al. 2000; Kennedy et al. 1998; Shukla et al. 2000). Oxidative stress, in turn, may be involved in the mechanism of RA initiation after respiratory exposure.

This analysis has several important limitations. We used distance to A1–A3 road as a proxy for exposure, so we did not have information on actual pollutant levels at the residential addresses over time. Additionally, we do not have information on the intensity of traffic, which could be used as an additional proxy for the exposures experienced at these residential locations. However, the results were slightly stronger when we considered only primary roads, presumably with higher traffic volume. We did not have power to break out the road categories individually. In our primary analyses, we focused on the distance to road in 2000, because that was the year the Census TIGER file of roads was created (U.S. Census Bureau 1992). In our sensitivity analyses using other available address years (1986–2004), the results were similar, suggesting that this is not likely to be an important limitation, potentially because most of the cohort had only one address during this time period and results in nonmovers were also similar. By using only one point in time, however, we are unable to determine potentially important time windows of exposure. All of these issues likely lead to exposure misclassification, which would bias our results toward the null and make associations harder to detect. Our conclusions are also weakened by the lack of a dose response. It is also possible that access to rheumatology specialists and differences in RA diagnostic proclivity may be an unmeasured confounder in this study. We examined differences in the RA cases at diagnosis according to distance to road category, and although the mean age at diagnosis did not vary with distance to road, the percentage of RF-positive cases increased at greater distances.

## Conclusions

The observed association between exposure to traffic pollution and RA suggests that pollution from traffic in adulthood may be a newly identified environmental risk factor for RA. This association study should be followed by further research into whether fine particles or chemicals are associated with RA risk and to discern whether there are time windows of exposure that may be particularly important.

## Figures and Tables

**Table 1:** Age-standardized characteristics of the study population (*n* = 90,297) at baseline (1976) by distance to road category.

	Distance to A1–A3 road in 2000 (m)		
Characteristic	0 to < 50	≥ 50 to < 200	≥ 200
No. (%)	5,183	10,264	74,850
Age [years (mean)]	42.8	42.8	42.3
BMI (mean)	24.1	23.9	23.7
Age (years) at menarche (mean)	12.4	12.5	12.4
Pack-years of smoking (mean)^a^	18.5	18.4	17.4
Caucasian race (%)	94.7	93.4	93.5
Smoking status (%)			
Current	31.3	33.7	31.1
Former	22.8	23.1	24.1
Never	45.7	43.1	44.5
Parity/lactation (%)			
Nulliparous	6.7	7.2	6.8
Parous, never breast-fed	31.4	33.0	30.0
Parous, breast-fed 1–11 months	36.0	35.0	37.0
Parous, breast-fed ≥ 12 months	15.9	14.0	15.8
Menopausal status (%)			
Premenopausal	66.7	66.3	67.8
Postmenopausal	29.8	29.6	28.4
Unknown status	3.6	4.2	3.9
Postmenopausal hormone use (%)^b^			
Never used	52.2	54.8	53.3
Past use	17.9	18.1	17.9
Current use	26.7	24.0	25.7
Oral contraceptive use (%)			
Never used	54.0	54.0	50.7
Ever use	46.0	46.0	49.3
Physical activity (metabolic equivalent hours/week, %)			
< 3	21.3	20.7	20.3
3 to < 9	20.6	20.8	19.8
9 to < 18	14.0	13.4	14.6
18 to < 27	7.9	7.5	8.0
≥ 27	10.9	11.2	11.2
Father’s occupation (%)			
Professional/manager	22.9	24.9	25.8
Other job	77.2	75.1	74.3
Mother’s occupation (%)			
Housewife	64.7	63.7	64.1
Other job	35.3	36.4	35.9
Education (%)			
Nurse	76.6	76.1	76.6
Other	23.5	23.9	23.4
Marital status (%)			
Married	65.1	63.8	67.3
Other	20.2	21.4	17.9
Husband’s education (%)			
Missing or not applicable	31.4	33.1	31.1
< high school	6.0	4.9	3.9
High school	31.6	28.3	26.6
> high school	31.1	33.8	38.5
Median census tract family income ($, mean)	57,419	60,657	64,431
Median census tract household value ($, mean)	142,796	159,929	174,297

a Among ever-smokers.

b Among postmenopausal women.

**Table 2:** HRs (95% CIs) from Cox proportional hazard models for incident RA (1976–2004) with distance to the closest A1–A3 road measured in 2000.

Distance to road (m)	Cases	Person-years	Basic model^a^	Adjusted model^b^
Whole cohort (*n* = 90,297)				
0 to < 50	52	136,205	1.33 (1.00–1.77)	1.31 (0.98–1.74)
≥ 50 to < 200	67	271,200	0.85 (0.66–1.09)	0.84 (0.65–1.08)
≥ 200	568	1,976,600	Reference	Reference
Nonsmokers (*n* = 40,128)^c^				
0 to < 50	23	62,223	1.63 (1.06–2.52)	1.62 (1.04–2.52)
≥ 50 to < 200	21	117,291	0.79 (0.50–1.24)	0.78 (0.49–1.22)
≥ 200	200	883,025	Reference	Reference

a Adjusted for current age in months and calendar year.

b Adjusted for current age in months, race, smoking status (current, former, never), pack-years of cigarette smoking, age at menarche, parity, duration of lactation, menopausal status, menopausal hormone use, oral contraceptive use, race, physical activity, body mass index, mother’s and father’s occupation, education level, martial status, husband’s education, and census-tract-level median family income and house value.

c Never-smokers at baseline (1976).

**Table 3:** HRs (95% CIs) from Cox proportional hazard models for incident RA (1976–2004) with distance to the closest A1–A3 road measured in 2000 by RF status.

Distance to road (m)	Cases	Person-years	Basic model^a^	Adjusted model^b^
RF-positive RA				

Whole cohort (*n* = 90,297)				
0 to < 50	32	136,221	1.45 (1.01–2.09)	1.44 (1.00–2.07)
≥ 50 to < 200	36	271,227	0.80 (0.57–1.13)	0.79 (0.56–1.12)
≥ 200	322	1,976,812	Reference	Reference
Nonsmokers (*n* = 40,128)^c^				
0 to < 50	12	62,234	1.51 (0.83–2.75)	1.51 (0.82–2.77)
≥ 50 to < 200	10	117,300	0.68 (0.35–1.29)	0.65 (0.34–1.26)
≥ 200	113	883,093	Reference	Reference

RF-negative RA				

Whole cohort (*n* = 90,297)				
0 to < 50	20	136,231	1.17 (0.74–1.85)	1.15 (0.73–1.83)
≥ 50 to < 200	31	271,233	0.91 (0.62–1.32)	0.90 (0.62–1.31)
≥ 200	246	1,976,865	Reference	Reference
Nonsmokers (*n* = 40,128)^c^				
0 to < 50	11	62,234	1.79 (0.95–3.37)	1.77 (0.93–3.38)
≥ 50 to < 200	11	117,299	0.94 (0.50–1.77)	0.88 (0.46–1.69)
≥ 200	87	883,133	Reference	Reference

a Adjusted for current age in months and calendar year.

b Adjusted for current age in months, race, smoking status (current, former, never), pack-years of cigarette smoking, age at menarche, parity, duration of lactation, menopausal status, menopausal hormone use, oral contraceptive use, race, physical activity, body mass index, mother’s and father’s occupation, education level, martial status, husband’s education, and census-tract-level median family income and house value.

c Never-smokers at baseline (1976).

**Table 4:** HRs (95% CIs) from Cox proportional hazard models for incident RA (1976–2004) with distance to the closest A1–A2 road measured in 2000.

Distance to road (m)	Cases	Person-years	Basic model^a^	Adjusted model^b^
Whole cohort (*n* = 90,297)				
0 to < 50	22	44,674	1.68 (1.10–2.58)	1.63 (1.06–2.51)
≥ 50 to < 200	32	127,147	0.87 (0.60–1.24)	0.86 (0.60–1.23)
≥ 200	633	2,224,161	Reference	Reference
Nonsmokers (*n* = 40,128)^c^				
0 to < 50	5	19,116	1.12 (0.46–2.72)	1.12 (0.46–2.75)
≥ 50 to < 200	11	54,525	0.86 (0.47–1.57)	0.86 (0.46–1.58)
≥ 200	228	994,300	Reference	Reference

a Adjusted for current age in months and calendar year.

b Adjusted for current age in months, race, smoking status (current, former, never), pack-years of cigarette smoking, age at menarche, parity, duration of lactation, menopausal status, menopausal hormone use, oral contraceptive use, race, physical activity, body mass index, mother’s and father’s occupation, education level, martial status, husband’s education, and census-tract-level median family income and house value.

c Never-smokers at baseline (1976).
